# Fellow-Workers and Their Doings

**Published:** 1914-02-28

**Authors:** 


### ROUND THE WORLD.

### Fellow-Workers and their Doings.

[The Editor Invites Early Information and Contributions Marked "Fellow-Workers."]

Dr. G. T. Western and Dr. Benians are working in the bacteriological laboratories of the London Hospital on problems of puerperal sepsis, in reference to which it may be called to mind that the death-rate from this condition is, so far as can be estimated, about two per 1,000, and has remained at this figure, or thereabouts, for some years. Dr. Western's observations have demonstrated that vaccines prepared from organisms found in the placental site are efficacious in some instances of puerperal septicæmia; the necessary material for cultivation being obtained under an anæsthetic.

Dr. Western, M.D.Cantab., has given special attention to bacteriology ever since he qualified from the "London" in 1905, and has during the last three or four years published important papers on his researches on puerperal septicæmia. He is the senior assistant in the inoculation department at the London Hospital.

Dr. Benians, M.R.C.S., L.R.C.P., is junior assistant in the inoculation department at the "London," and has done good work in the development of technical bacteriological procedures; he is now investigating the bacteriology of acne.

The successful treatment by diathermy at St. Bartholomew's Hospital, the progress of which was recently referred to in The Hospital (February 7), is under the direction of Dr. E. P. Cumberbatch, M.B., M.R.C.P., who has lately been directing the electro-therapeutic department developed by the keen efforts of Dr. Lewis Jones. Dr. Cumberbatch is an old pupil of Gotch and Burdon Sanderson, whose memory will be for ever preserved in their eletcro-physiological records. He will probably sum up his results on diathermy at the forthcoming British Medical Association meeting at Aberdeen.

The new occupant of the Walter Myers Professorship of Parasitology in Liverpool University, Dr. Warrington Yorke, has never lost touch with his *Alma Mater*, whose degrees in medicine and surgery are his medical qualifications. Having served as house surgeon and house physician to the Royal Infirmary at Liverpool, Professor Yorke became Holt Fellow in Physiology at his University and director of the Runcorn Research Laboratory of the Liverpool School of Tropical Medicine; he also served with the Black-water Fever Expedition sent to Central Africa by that school in 1907.

Surgeon-General W. Burney Bannerman, who has just been appointed to the vacancy in the list of honorary physicians to H.M. the King, a coveted military distinction, has had a distinguished career in the Indian Medical Service. He received his medical training at Edinburgh University, where he took the M.D. degree in 1889. Surgeon-General Bannerman has done a great deal of work in connection with plague and the inoculation treatment thereof; whilst among other important posts he has held that of director of the Bombay Bacteriological Laboratory.

Dr. Kenneth D. Wilkinson, who has been appointed head of the pathological department at the Birmingham and Midland Free Hospital for Sick Children, is an M.D., Ch.B. of Birmingham University, and has published numerous papers on the pathology and clinical phenomena of heart block.

Another ophthalmic registrarship has fallen vacant at "Guy's" owing to the appointment of its recent holder, Mr. George Maxted, M.D., B.S.Lond., as assistant surgeon to the Norwich and Norfolk Eye Infirmary. Mr. Maxted has held several resident appointments at "Guy's," and was successful in obtaining the gold medal in obstetric medicine at the M.D.Lond. examination two years ago.

Dr. John Carswell, F.F.P.S.Glasg., L.R.C.P.Edin., who has lately been appointed one of his Majesty's Scottish Commissioners in Lunacy, has served in several important offices. Among these may be mentioned the posts of physician to the department for nervous and mental diseases at Glasgow Public Dispensary; physician to the mental department at the East District Hospital, Glasgow; medical officer for mentally defective children under the Glasgow School Board; and lecturer on mental diseases at Anderson's College Medical School, Glasgow.

The vacant registrarships in the ophthalmic department at Guy's Hospital have been filled by the election of Mr. C. M. Ryley and Mr. J. S. Bookless, M.B., B.S.Lond., who has lately been clinical assistant in the department as well as clinical assistant at the Royal London Ophthalmic Hospital.

Mr. W. A. Wigram has accepted temporarily the office of acting honorary secretary and treasurer to the Walton, Hersham, and Oatlands Cottage Hospital, owing to the retirement for the present of Mr. Edward Pettit on the unfortunate ground of ill-health. Mr. Pettit has been an active worker for the cottage hospital since it was opened in 1905.

Another hospital honorary secretary who has been compelled to give up work is Mr. J. E. W. Wakefield, whose illness has interrupted his labours for the Taunton and Somerset Hospital. He has been prevailed on to withhold his resignation, and as he is understood to be making good progress, his colleagues hope to have him back again in active harness before very long.

Mr. Cardross Grant, though he has given up the hon. secretaryship of Beckenham Cottage Hospital, retains the treasurership, and his previous share of this formerly joint office is being undertaken by Mr. Crowther Benyon.

Dr. E. B. Sherlock, the new superintendent of the Darenth Industrial Colony (Metropolitan Asylums Board), has lately been acting superintendent of the Fountain Hospital at Tooting.

